# A national survey of PICU clinician practices and perceptions about respiratory cultures for invasively ventilated patients

**DOI:** 10.1017/ash.2023.236

**Published:** 2023-09-29

**Authors:** Anna Sick-Samuels, Danielle Koontz, Anping Xie, Daniel Kell, Charlotte Woods-Hill, Anushree Aneja, Shaoming Xiao, Elizabeth Colantuoni, Jill Marsteller, Aaron Milstone

## Abstract

**Background:** Respiratory cultures are commonly obtained from patients with suspicion for ventilator-associated infections (VAIs). In the absence of specimen ordering and collection guidelines, management practices may differ. We characterized current respiratory culture collection practices and perceptions and identified potential barriers to changing practices among a national collaborative of pediatric intensive care units (PICUs). **Methods:** We conducted an electronic survey of PICU physicians, advanced practice providers (APPs), respiratory therapists (RTs), and nurses at 16 US academic pediatric hospitals across the United States. Positive Likert-scale responses (eg, “agree” and “strongly agree”) were grouped. To account for varying hospital representation, we analyzed the results as the median proportion of participants with that response across the hospitals. **Results:** After excluding incomplete responses, 568 (44%) of 1,301 invited participants responded (range, 16–107 per site); the median hospital response rate was 60% (range, 17%–83%). Roles included physicians (35%), APPs (10%), RTs (24%), and nurses (31%). Moreover, 44% of the participating units cared for cardiac surgery patients. Across hospitals, specimens are often collected by RTs, followed by nurses, typically via inline endotracheal aspirate for either endotracheal tubes or tracheostomies. Saline lavage is a common practice, but only 4% reported a standardized approach. Examining the likeliness to obtain cultures for different clinical symptoms, the widest variation in responses were for fever and inflammatory markers without respiratory symptoms (median proportion, 68%; IQR, 54%–79%), isolated change in secretion characteristics (67%; IQR, 54%–78%), isolated increased secretions (55%; IQR, 40%–65%), isolated inflammatory markers (49%; IQR, 38%–57%) or isolated fever (49%; IQR, 38%–61%). Overall, 75% (IQR, 70%–86%) of reported respiratory cultures were likely to be obtained as a “pan culture.” Most respondents (median proportion, 69%) felt confident about the indications to obtain cultures, but 60% felt that clinicians had a low threshold, and 84% reported clinical practice variation. Barriers to change included reluctance to change (70%), opinion of consultants (64%), and fear of missing a diagnosis of VAI (62%). Respondents agreed that they would find clinical decision support (CDS) tools helpful (79%). In addition, 83% expected that they would follow CDS, and 82% thought that CDS would help align ICU and/or consulting teams. **Conclusions:** Among 16 participating hospitals, we detected a lack of standardized respiratory-culture specimen collection and ordering practices. Most respondents agreed that CDS tools would be helpful. Diagnostic stewardship of respiratory cultures using CDS must account for potential reluctance to change and needs to address stakeholder perspectives, including fear of missing infections.

**Disclosure:** None

